# *In Vitro* and *In Vivo* Evaluation of Cysteine and Site Specific Conjugated Herceptin Antibody-Drug Conjugates

**DOI:** 10.1371/journal.pone.0083865

**Published:** 2014-01-14

**Authors:** Dowdy Jackson, John Atkinson, Claudia I. Guevara, Chunying Zhang, Vladimir Kery, Sung-Ju Moon, Cyrus Virata, Peng Yang, Christine Lowe, Jason Pinkstaff, Ho Cho, Nick Knudsen, Anthony Manibusan, Feng Tian, Ying Sun, Yingchun Lu, Aaron Sellers, Xiao-Chi Jia, Ingrid Joseph, Banmeet Anand, Kendall Morrison, Daniel S. Pereira, David Stover

**Affiliations:** 1 Agensys, Inc, Santa Monica, California, United States of America; 2 Ambrx Incorporated, La Jolla, California, United States of America; National Cancer Institute, NIH, United States of America

## Abstract

Antibody drug conjugates (ADCs) are monoclonal antibodies designed to deliver a cytotoxic drug selectively to antigen expressing cells. Several components of an ADC including the selection of the antibody, the linker, the cytotoxic drug payload and the site of attachment used to attach the drug to the antibody are critical to the activity and development of the ADC.

The cytotoxic drugs or payloads used to make ADCs are typically conjugated to the antibody through cysteine or lysine residues. This results in ADCs that have a heterogeneous number of drugs per antibody. The number of drugs per antibody commonly referred to as the drug to antibody ratio (DAR), can vary between 0 and 8 drugs for a IgG_1_ antibody. Antibodies with 0 drugs are ineffective and compete with the ADC for binding to the antigen expressing cells. Antibodies with 8 drugs per antibody have reduced *in vivo* stability, which may contribute to non target related toxicities.

In these studies we incorporated a non-natural amino acid, para acetyl phenylalanine, at two unique sites within an antibody against Her2/neu. We covalently attached a cytotoxic drug to these sites to form an ADC which contains two drugs per antibody.

We report the results from the first direct preclinical comparison of a site specific non-natural amino acid anti-Her2 ADC and a cysteine conjugated anti-Her2 ADC. We report that the site specific non-natural amino acid anti-Her2 ADCs have superior *in vitro* serum stability and preclinical toxicology profile in rats as compared to the cysteine conjugated anti-Her2 ADCs. We also demonstrate that the site specific non-natural amino acid anti-Her2 ADCs maintain their *in vitro* potency and *in vivo* efficacy against Her2 expressing human tumor cell lines. Our data suggests that site specific non-natural amino acid ADCs may have a superior therapeutic window than cysteine conjugated ADCs.

## Introduction

Antibody-drug conjugates (ADCs) are antibodies engineered to deliver a cytotoxic drug directly to tumor cells expressing the appropriate cell surface antigen. The selective and stable delivery of the cytotoxic drug to the tumor and not to the normal tissues should reduce the toxicities associated with cytotoxic drug and potentially improve the therapeutic index of the ADC. Successful development of an ADC involves optimization of several components including the antibody, the potency of the cytotoxic drug, the stability of the linker and the site of drug-linker attachment [1]. In order to begin our evaluation, we selected the clinically validated antibody, Herceptin, for our studies.

Herceptin® (Trastuzumab) is a humanized IgG_1_ monoclonal antibody that binds to human Her2/neu, which is highly expressed on breast, ovarian and gastric cancers [2]. Amplification of Her2/neu results in increased Her2/neu expression and is associated with a poor prognosis [2], [3].

Herceptin was approved by the United States Food and Drug Administration (FDA) in 1998 for the treatment of metastatic breast cancer. In 2010 Herceptin was also approved by the FDA for the treatment of Herceptin expressing metastatic cancer of the stomach or gastroesophageal junction. Herceptin, when combined with chemotherapy, has provided substantial benefits to patients in the form of improved progression free survival and overall survival [4], [5]. One of the problems commonly associated with treating cancer patients is the tumors either have intrinsic resistance or develop an acquired resistance to treatment over time. Resistance to Trastuzumab has been reported in patients who were previously treated with Trastuzumab or Lapatinib. Several mechanisms of resistance to Trastuzumab have been proposed to explain how tumors become resistant to Trastuzumab but none have been validated clinically [6]. Interestingly, preclinical studies have shown that treating Trastuzumab-resistant tumors with a Trastuzumab ADC can inhibit the growth of Trastuzumab-resistant tumors [7].

A lysine conjugated ADC comprised of Trastuzumab and the maytansinoid drug payload, N(2′)-deacetyl-N(2′)-(3-mercapto-1- oxopropyl)-maytansine (DM1), which is also known as Ado-Trastuzumab Emtansine (T-DM1), has recently been approved for the treatment of Her2 positive breast cancer patients [8]. Recent clinical data show a 9.6 month median progression free survival (PFS) for breast cancer patients treated with T-DM1 compared to 6.4 months for patients treated with Tykerb (lapatinib) and Xeloda (capecitabine) [9]. These data suggest that T-DM1 may offer a significant survival advantage over the current standard of care agents for Her2 positive breast cancer patients.

The conjugation of DM1 on lysine residues results in a heterogeneous distribution of antibodies which contain 0 to 8 drugs per antibody, with an average of 3.5 drugs per antibody. In order to produce an ADC with a homogeneous number of drugs per antibody, the amino acid Alanine at position 114 (Ala 114), on the antibody heavy chain, was modified to a cysteine. The drug payload, DM1 was conjugated to the thiol on the cysteine, which resulted in the homogenous incorporation of two DM1 molecules per antibody (ThioTMab). ThioTMab was reported to have comparable efficacy in preclinical tumor models as T-DM1. Toxicology studies, which were carried out in rats and cynomolgus monkeys, reported that ThioTMab was better tolerated in rats and monkeys as compared to T-DM1 [10].

In this study we incorporated the non-natural amino acid, p-acetyl phenylalanine (pAF), site specifically into the heavy chain of an antibody against Her2/neu. The amino acid, Ala 114, on the anti-Her2 antibody's heavy chain, was replaced with pAF. A second distinct site of conjugation was also evaluated, denoted as position 2, but is not disclosed. The ADC drug payload, denoted as payload A, was conjugated to pAF containing antibodies via an oxime bond, which produces a homogeneous distribution of approximately 2 drugs per antibody[11]. The site specific non-natural amino acid containing anti-Her2 ADCs or anti-CD22 ADCs are described as anti-Her2 position 1-ADC, anti-Her2 position 2-ADC, anti-CD22 position 1-ADC or anti-CD22 position 2-ADC.

The second ADC drug payload, denoted as payload B, was conjugated to cysteine residues, via thiols, on the non pAF containing antibodies. This conjugation produces a heterogeneous distribution of drugs per antibody with an average of 3.8 drugs per antibody. The cysteine conjugated anti-Her2 or anti-CD22 ADCs are described as anti-Her2 Cys-ADC or anti-CD22 Cys-ADC.

Our study is the first where site specifically conjugated; non-natural amino acid containing ADCs and a cysteine conjugated ADC have been directly compared for *in vitro* cytotoxicity, *in vivo* tumor efficacy, pharmacokinetic properties and rat toxicology have evaluated within the same study. Furthermore we are providing a direct comparison of the Chemical, Manufacturing and Control (CMC) properties and the *in vitro* antibody serum stability of the cysteine conjugated ADCs and the site specifically conjugated; non-natural amino acid containing ADCs.

## Materials and Methods

### Ethics statement

All studies conducted in laboratory animals described in this manuscript were carried out in strict accordance with the applicable sections of the Guide for the Care and Use of Laboratory Animals from the National Research Council. The protocol and any amendments or procedures involving the care or use of animals in these studies were reviewed and approved by Agensys or Charles River Institutional Animal Care and Use Committee before the initiation of such procedures. All efforts were made to minimize pain and suffering. If an animal was determined to be in overt pain/distress, or appeared moribund and was beyond the point where recovery appeared reasonable, the animal was euthanized for humane reasons in accordance with the AVMA Guidelines on Euthanasia.

### Expression and purification of antibodies with non-natural amino acids

Antibodies with site specific incorporation of pAF were expressed as previously described in Axupa et al, 2012 [11]. Antibodies were purified over a protein A column (NovaSep) followed by a SP 650S column (Tosoh Biosciences).

### Synthesis of the ADC payloads and linkers

The drug payloads, payload A and payload B, and drug linkers were synthesized as previously described in Axupa et al, 2012 [11].

### Site specific conjugation

Para-acetyl phenylalanine containing anti-Her2 antibodies were buffer exchanged into 50 mM sodium acetate, pH 4.0; 1% acetic hydrazide to a final concentration of 10 mg/ml. 10 molar equivalents of hydroxyl-amine drug-linker (payload A) was added and reacted for 18 hours at 28°C. The antibody conjugates were purified over a SP 650S column (Tosoh Biosciences) to remove excess reagents. The conjugates were buffer exchanged into 50 mM histidine, 100 mM NaCl, 5% trehalose, pH 6.0, filtered, and stored at ≤65°C.

### Intact mass determination

Intact mass analysis was performed on deglycosylated anti- Her2 antibody, unconjugated site specific anti-Her2 antibody and conjugated site specific anti-Her2 ADC. The samples were treated with PNGase overnight at 37°C in 0.1M tris pH 8.5 followed by reduction in 4M guanidine-HCl, 0.1M tris pH 8.0, 0.05M DTT for 10 min at 85°C. The reduced deglycosylated samples were analyzed for intact mass on a Dionex 3000 UHPLC separation system in tandem with and Orbitrap Velos (Thermo Scientific, West Palm Beach, FL). Samples were applied onto a POROS R2 10 µm column, 2.1× 100 mm (Applied Biosystems, Carlsbad, CA). The column was equilibrated in 75% mobile phase A (0.1%TFA in water) and 25% mobile phase B (0.1% TFA in 90% acetonitrile/10% HPLC H_2_O) with a gradient of 2.5%/min over 10 minutes with a flow rate of 0.3 mL/min. Column temperature was set to 70°C.

Intact mass spectra were collected in a positive ion mode with the mass range set to 700–3000 m/z with capillary and source temperatures at 300°C, sheet gas flow rate at 50 arbitrary units and auxiliary gas flow rate at 8 arbitrary units. The capillary voltage was set to 3500V with the fragmentor voltage set to 75V. The acquired spectra were deconvoluted using Thermo Deconvolution 2.0 software.

### Preparation of the Anti-Her2 Cys-ADCs

The anti-Her2 antibody buffer was exchanged to a final concentration of 8 mg/mL in 5 mM EDTA, 20 mM Tris, pH 7.4 was added 2.3 molar equivalent of TCEP and incubated at 37°C for 2 hr. Payload B in 8% DMSO, 4.8 molar equivalents were added to the reaction mixture and reacted for 1 hr at room temperature. The reaction was quenched by adding 4.8 molar equivalents of N-Acetylcysteine. The anti-Her2-Cys-ADC was purified by using PD-10 columns with elution of 20 mM Histidine, 5% sucrose, pH 6.0 buffer.

### Preparation of the Anti-CD22 Cys-ADC

The anti-CD22 Cys-ADC was prepared using the same methodology as the anti-Her2 Cys-ADC.

### DAR determination and antibody preparation evaluation

The drug antibody ratio (DAR) was determined under denaturing and reducing conditions using the reverse phase HPLC column (2.1×50 mm 8 µm PLRP-S column, Agilent Technology). The amount of unconjugated antibody was determined using the hydrophobic interaction column (TSKgel Butyl-NPR, 4.6 mm×3.5 cm, 2.5 m, Tosoh Bioscience). The amount of aggregation was determined by size exclusion HPLC (TSKgel 3000SW, 7.8 mm×300 mm, Tosoh Bioscience).

### Cell culture conditions

The HCC-1954, NCI-N87, MDA-MB-468 and MDA-MB-453 cell lines were purchased from the ATCC. The HCC-1954, MDA-MB-453 and NCI-N87 cells were maintained in RPMI-1640 culture media supplemented with 10% FBS in a humidified incubator set at 5% CO_2_ and 37°C. The MDA-MB-468 cells were maintained in L-15 culture media supplemented with 10% FBS in a humidified incubator set at 0% CO_2_ at 37°C.

### Determination of cell surface Her2 expression

The Her2 cell surface expression was evaluated on HCC-1954, NCI-N87, MDA-MB-468 and MDA-MB-453 human tumor cell lines via FACS analysis. Briefly, cells were harvested and incubated for 1 hour at 4°C with the anti-Her2 antibody, Herceptin or an isotype control antibody (10 µg/ml) in PBS+2% FBS. The cells were washed and incubated with anti-hIgG-PE antibody (Jackson ImmunoResearch Laboratories, Inc) at a 1∶100 dilution in PBS+2% FBS for 1 hour at room temperature. The cells were washed to remove any unbound antibody and suspended in PBS for analysis. Fluorescence was measured by the Attune cytometer and the relative mean fluorescence intensity (MFIR) values for each sample are included in the representative graph.

### Antibody cell surface affinity

The affinity of the anti-Her2 and anti-CD22 antibodies was determined by flow cytometry. Briefly, 50 µL of each prediluted mAb dilution curve was added to the 96-well plates containing 100 µL of either HCC-1954 or MDA-MB-468 cells. The plate was incubated at 4°C on a plate shaker overnight. Following the primary incubation, the cells were washed once with FACS buffer (FB). The FB was discarded and 100 µL of goat anti-human IgG-PE (Jackson ImmunoResearch Laboratories, Inc) secondary antibody (diluted 1∶100) was added to each well. The plate was incubated at 4°C for 1 hour. The cells were washed twice and re-suspended in 200 µL of FB. The plate was read on the FACScan flow cytometer (BD Biosciences).

Following data acquisition, the data was analyzed by first extracting the mean fluorescence intensity (MFI) for each sample using Cellquest Pro (BD Biosciences). The MFI values were then imported into Graphpad Prism (GraphPad Software, Inc.) for further analysis. The data was graphed using the MFI values versus the test sample concentration (nM). Detectable binding of antibodies over the range of test concentrations (MFI values above background) were analyzed using the one-site binding, non-linear regression analysis. The binding K_D_ (equilibrium dissociation constant) and B_max_ (maximum specific binding) are reported.

### *In vitro* cytotoxicity analysis

The plating density and assay time course was optimized for each cell line. The cells were plated at an optimum density in a total of 50 µL of the appropriate culture media. The cells were allowed to attach to the plates overnight. Anti-Her2 ADCs, anti-CD22 ADCs or unconjugated antibodies were diluted to 10 µg/mL in culture media and serially diluted 1∶5 for a total of 9 points. The cells were treated with 50 µL of the diluted antibodies, which yielded a final concentration of 5000, 1000, 200, 40, 8, 1.6, 0.32, 0.064 and 0.0128 ng/mL. The cells were incubated with the diluted antibodies for 6 days at 37°C. The number of viable cells was evaluated by incubating the cells with 20 µL of Resazurin based Presto Blue® Cell Viability Reagent (Life Technologies) for 1 hr at 37°C. Fluorescence (540,590 nm) was measured using a Synergy H4 hybrid reader (BioTek). Three well replicate readings were obtained per treatment concentration, and the media only reading was subtracted from all wells. Untreated control cells were used to determine 100% survival. The percent of viable cells was calculated using the following formula <((txt-media)/(no txt-media))*100> and values used to calculate an EC_50_ value using Prism 5.04 by transforming concentrations to x = log(X) and fitting with a sigmoidal variable slope dose response.

### Immunohistochemical evaluation of Her2 expression in Formalin fixed tissue sections

Immunohistochemical evaluation of Her2 expression on formalin fixed paraffin embedded sections of HCC-1954 and NCI-N87 human tumor xenografts was performed using the Leica Bond Max autostainer (Leica Biosystems, Buffalo Grove, IL). After dewaxing and rehydration, the sections were treated with Bond Epitope Retrieval 2 solution and incubated with a 1∶200 dilution of Rabbit anti-Her2 antibody (Clone:SP3,Thermo-Scientific, RM-9103-S) or a non specific rabbit IgG (5 µg/ml). The sections were developed using the Bond Polymer Refine Detection system (Leica Biosystems, Buffalo Grove, Il). The sections were then counterstained in hematoxylin, washed, dehydrated and coverslips were applied.

### *In vivo* tumor efficacy studies

Female ICR SCID mice 6–8 weeks of age, with an average weight of approximately 25 grams, were purchased from Taconic Farms (Oxnard, CA). The mice were housed in ventilated cage racks. Food and water were provided *ad libitum*. The mice were acclimated for 72 hours before the studies were initiated.

Routine husbandry and handling was performed with experimental animals in compliance with regulations and guidelines governing the use of animals in research. All routine and the experimental animal work was conducted in compliance with the Agensys Institutional Animal Care and Use Committee (IACUC). Protocol #002 was approved prior to the commencement of the study. Study animals were humanely euthanized when the tumor volume reached 2000 mm^3^ or when they became moribund or showed signs of distress.

Two *in vivo* tumor efficacy studies were performed to evaluate anti tumor activity of the site specific and cysteine conjugated ADCs.

The HER2 expressing HCC-1954 breast cancer cell lines (3×10^6^ cells per mouse) or NCI-N87 gastric cancer (2×10^6^ cells per mouse) cells were resuspended in 200 µl of culture media containing 50% Complete Cultrex Matrix (Trevigen, Gaithesberg, MD). The HCC-1954 cells were inoculated into the right mammary fat pad of ICR female SCID mice, while the NCI-N87 cells were inoculated subcutaneously into the right flank of ICR male SCID mice. The tumor growth rate was monitored twice weekly, using caliber measurements. When the majority of tumors reached a predetermined size, the mice were assigned to treatment and control groups (n = 10 mice/group). The tumors were size matched across all groups. The health status of the mice was monitored daily and body weights were measured twice weekly using a digital scale.

The HCC-1954 tumor bearing mice were treated intravenously (i.v.) with a single injection of anti-Her2 position 1-ADC (at 0.5, 1 or 3.3 mg/kg) or anti-Her2 position 2-ADC (at 0.5 or 1 mg/kg) or anti-Her2 Cys-ADC (at 0.5, 1 or 3.3 mg/kg) or the control ADCs (Anti-CD22 Position 1-ADC or Anti-CD22 Cys-ADC at 3.3 mg/kg) or Vehicle (5% Dextrose) when the average tumor size reached approximately 200 mm^3^.

The NCI-N87 gastric tumor bearing mice were treated with a single i.v. injection of anti-Her2 position 1-ADC (1, 3 or 5 mg/kg) or anti-Her2 position 2-ADC (1, 3 or 5 mg/kg) or anti-Her2 Cys-ADC (1, 3 or 5 mg/kg) or anti-CD22 position 1-ADC, at 5 mg/kg or Vehicle (5% Dextrose) when the average tumor size reached approximately 200 mm^3^.

The *in vivo* tumor efficacy of the ADCs was evaluated by comparing the tumor volume of the control groups with the tumor volume of the treated groups. The mean tumor volume data for each group was plotted over time with standard error bars. Additionally, individual tumor volume data for the last day before animal sacrifice was plotted along with the mean and standard error bars to examine the distribution of the data.

A statistical analysis of the tumor volume data for the day before animal sacrifice was performed using the Kruskal-Wallis test. The implementation of the Kruskal-Wallis test was carried out using the parametric ANOVA F-test on the ranks of the data [12]. Pairwise comparisons were made using the Tukey–Kramer method (2-sided) to protect the experiment-wise error rate.

The p-value for each individual comparison is reported when p<0.05. The percent tumor growth inhibition in each treated group versus a control group was calculated as [(Control - Control baseline) - (Treated - Treated baseline)]/(Control - Control baseline)×100%. When tumors in a treated group showed regression (i.e., tumor volume at the last day before animal sacrifice was less than the baseline volume, hence the percent growth inhibition was more than 100%), then the percent change from baseline tumor volume (percent of regression) for that treated group was reported instead of the percent inhibition. The percent of regression was defined as (Treated baseline-Treated)/Treated baseline ×100%. The percent tumor growth inhibition was calculated by the change in the mean treated tumor volume divided by the change in the mean control tumor volume multiplied by 100 and subtracted from 100%. Statistical significance was determined using the unpaired Student's t-test or the ANOVA where appropriate.

### Rat toxicity and toxicokinetic analysis

The in-life toxicological assessments of the anti-Her2 position 1-ADC and the anti-Her2 Cys-ADC were evaluated using CRL:CD®IGS male rats (n = 5/group) at Charles River Laboratories (Reno, NV). The rats were given a single i.v. injection of the anti-Her2 Cys-ADC (30, 45, and 60 mg/kg) or the anti-Her2 position 1-ADC (30, 60 and 90 mg/kg). One group of rats (n = 5) was administered the vehicle. The in-life toxicological assessments included daily clinical observations, twice weekly body weight measurements, weekly measurements of food consumption and the clinical pathology (hematology and serum chemistry) on study days 3, 8 and 15 was reported. On day 15 the main study animals were euthanized. A full necropsy was performed. The organ weights were collected and histopathologic examination of the administration site, bone marrow, epididymis, heart, kidney, liver, lungs, spleen, testis and urinary bladder was conducted.

Satellite groups, which contained 4 rats per group, were also given a single i.v. injection of anti-Her2 Cys-ADC or anti-Her2 position 1-ADC. Blood was collected at 1, 24, 72 and 168 hours after the initial dose for determination of the toxicokinetic parameters and serum was isolated. A quantitative sandwich enzyme linked immunoassay (ELISA) was used to measure the amount of total anti-Her2 antibody and the amount of ADC in the serum samples.

This study was conducted in accordance with all applicable sections of the Final Rules of the Animal Welfare Act regulations (Code of Federal Regulations, Title 9), the *Public Health Service Policy on Humane Care and Use of Laboratory Animals* from the Office of Laboratory Animal Welfare, and the *Guide for the Care and Use of Laboratory Animals* from the National Research Council.

### Sample preparation for *in vitro* ADC serum stability studies and drug payload transfer to Human serum albumin (HSA)

To evaluate the *in vitro* serum stability of the ADCs and to measure the amount of drug payload transferred to HSA, the ADCs (100 µg/ml) were incubated with either 100% pooled human serum at 4°C or at 37°C for 0, 4 hr, 8 hr, 1, 3, 7, 14, 21, and 28 days or incubated with HSA (40 mg/ml) at 37°C for 0, 7, 14, and 28 days. The serum samples were stored at −70°C before they were analyzed for the amount of total antibody, ADC and the amount of drug payload transfer to HSA. The samples prepared for the HSA drug payload transfer assay were incubated with protein A to deplete the anti-Her2 antibody and to reduce the possible interference of the anti-ADC antibody in the HSA transfer assay. After incubation with protein A, at room temperature for 2 hours, 98% of the anti-Her2 antibody was depleted.

### Analysis of total antibody, ADC and drug payload transfer to HSA

The amount of total antibody and ADC in rat serum samples and in samples prepared for the *in vitro* serum stability study was evaluated using a quantitative sandwich enzyme linked immunoassay (ELISA). Briefly, ELISA plates were coated with 1 µg/ml of recombinant human anti-Her2/ErbB2 antibody (Sino Biologicals, Inc) and blocked with ELISA blocking buffer. The plates with immobilized antigen (Her2/ErbB2) were incubated with standard, QC and samples to capture the anti-Her2 antibodies (both unconjugated and ADC). After washing to remove unbound antibodies, the bound total anti-Her2 antibody (naked+ADC) was detected with horseradish peroxidase (HRP) conjugated Goat anti-human-Fc-specific polyclonal antibody (Jackson ImmunoResearch). For the determination of anti-Her2 ADC only, a biotin-conjugated mouse antibody specific to Auristatin analogs (1.55 µg/ml) was used as the detection antibody, followed by addition of a streptavidin conjugated to HRP. After washing to remove unbound detection antibody, a slow kinetic substrate solution of tetramethylbenzidine (TMB) was added to the wells where color develops in proportion to the amount of anti-Her2 position 1-ADC or anti-Her2 Cys-ADC. The optical density (OD) of the color was measured at 650 nm and was used for determination of the amount of anti-Her2 antibody in the test samples by extrapolation from standard curves of the same test article. To determine drug payload to HSA transfer, mouse antibody specific to an Auristatin analog was used as the capture antibody, and detected with an anti-HSA-HRP antibody.

## Results

### LC/MS antibody characterization

**Figure 1:**
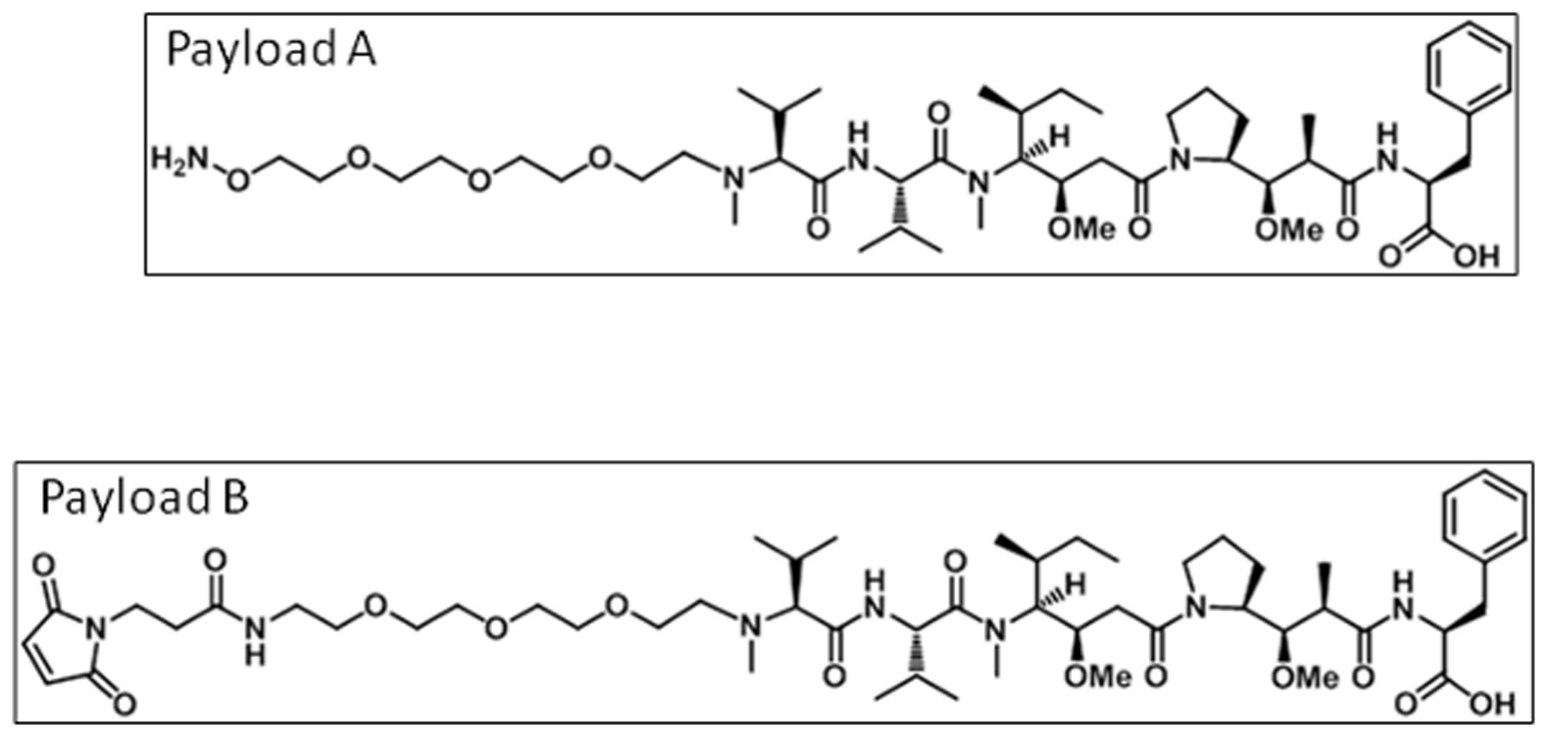
ADC payload chemical structures. The chemical structures of the ADC payloads are shown. Payload A was used to conjugate to pAF via an oxime bond. Payload B was used to conjugate to the thiols on cysteines via maleimide.

**Figure 2:**
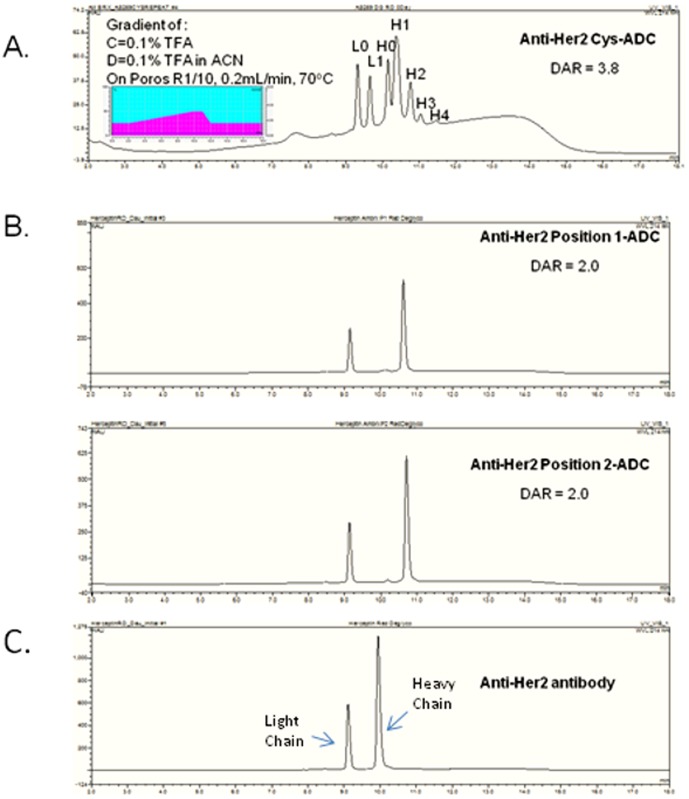
Antibody HPLC analysis. The reversed phase HPLC of deglycosylated reduced and denatured (A) Anti-Her2 Cys-ADC, (B) Anti-Her2 Position 1-ADC, Anti-Her2 Position 2-ADC and (C) Anti-Her2. Insert shows the gradient profile and chromatography conditions. There were two drugs/antibody determined by the intact MS analysis or one drug per heavy chain. The reversed phase HPLC profile of the cysteine conjugated anti-Her2 ADC shows the number of drugs conjugated to the heavy (H) and light (L) chains determined by the intact mass spectrometry analysis.

The conventional conjugation of drug payload B (Figure 1) to cysteines results in a heterogeneous mixture of drugs per antibody ranging from 0 to 8 drugs per antibody. The chromatographic profile for the cysteine conjugated anti-Her2 antibody (anti-Her2 Cys-ADC) shows a heterogeneous mixture of drug payload conjugated to the heavy and light chains with an average of 3.8 drugs per antibody (Figure 2A). In contrast the site specific conjugation of payload A (Figure 1) to pAF containing anti-Her2 antibodies, which has pAF incorporated in two specific locations on the heavy chain, has a homogeneous distribution of 2 drugs (payload A) per antibody (Figure 2B). The chromatographic profile of the unconjugated non-natural amino acid anti-Her2 antibody is displayed in Figure 2C. A shift in the molecular weights of the heavy chain of the anti-Her2 ADCs is observed when compared against the molecular weight of the unconjugated non-natural amino acid containing anti-Her2 antibody.

### *In vitro* ADC serum stability studies and the analysis of the drug payload transfer to HSA

**Figure 3:**
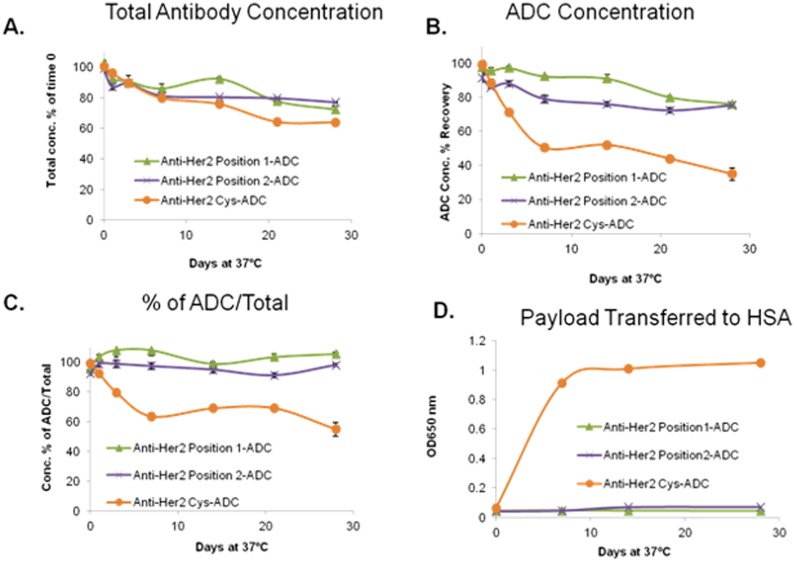
*In vitro* antibody stability. The anti-Her2 ADC antibodies were incubated in human serum or HSA at 37°C up to 28 days. (A) The amount of total antibody (naked and ADC) in the preparation was measured. (B) The amount of ADC in the preparation was measured. (C) % Concentration of ADC over the amount of total antibody. (D) The amount of drug conjugated HSA is measured.

As shown in Figures 3A and 3B, the concentration of total anti-Her2 antibody (unconjugated and ADC) and ADC were reduced in the cysteine conjugated anti-Her2 antibody after incubation with human serum at 37°C. A 20% decrease in the amount of total antibody was observed on day 7 and a 29% decrease in the amount of ADC was observed on day 3. On day 28, the decrease reached 36% for amount of total antibody and 65% for ADC. In contrast, anti-Her2 position 1-ADC and anti-Her2 position 2-ADC achieved a maximum decrease of approximately 25% on day 28 and there was no difference in the concentration between amount of total antibody and ADC for each time point (Figure3C). The data indicated that the cysteine conjugated anti-Her2 antibody was not stable, after incubation with human serum at 37°C, as compared to the site specific anti-Her2-antibody ADCs. Furthermore, the greater decrease in the ADC concentration indicated that the anti-Her2 Cys-ADC may lose its payload faster than the non-natural amino acid position 1 or position 2 anti-Her2 ADCs. To investigate the possibility of drug payload transfer to HSA, we monitored the presence of HSA drug conjugates. Our data show that the amount of HSA drug conjugates increases over time when the anti-Her2 Cys-ADC was incubated with HSA. No increase in the amount of HSA-drug conjugate was observed when the anti-Her2 position 1 or anti-Her2 position 2 ADCs were incubated with HSA (Figure 3D).

### Her2 expression and *In vitro* cytotoxicity

**Figure 4:**
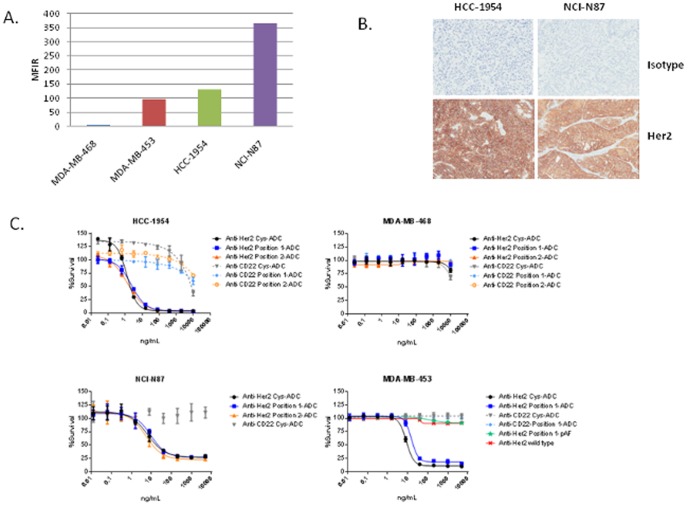
*In vitro* evaluation of Her2 expression and ADC cytotoxicity. The expression of Her2 and the *in vitro* cytotoxicity of the ADCs are evaluated in the HCC-1954, NCI-N87, MDA-MB-453 and MDA-MB-468 cell lines. (A) The cell surface expression of Her2 is measured via FACS for a panel of tumor cell lines. HCC-1954, MDA-MB-453 and NCI-N87 were selected as the Her2 expressing cell lines while MDA-MB-468 was selected as the Her2 negative cell line. MDA-MB-453 cells are resistant to Herceptin. (B) NCI-N87 and HCC-1954 tumor xenografts were evaluated for Her2 expression via IHC. (C) The *in vitro* cytotoxicity of the anti-Her2 ADCs were evaluated in the HCC-1954, NCI-N87, MDA-MB-453 and MDA-MB-468 cell lines.

The cell surface Her2 expression was evaluated by FACS analysis on the HCC-1954, NCI-N87, MDA-MB-453 and MDA-MB-468 tumor cell lines (Figure 4A). HCC-1954 and the NCI-N87 cell lines express similar levels of Her2 while MDA-MB-468 cells do not express Her2. Immunohistochemical analysis of Her2 expression on NCI-N87 and HCC-1954 tumor xenografts confirm that both tumors express Her2. Non specific binding of a control antibody to the tumor sections was not observed (Figure 4B).

The unconjugated pAF-containing anti-Her2 antibody (anti-Her2 position 1-pAF), the unconjugated native (wild type) anti-Her2 antibody and the conjugated anti-Her2 antibodies had similar binding affinities to cell surface Her2 (Table 1). The anti-CD22 antibodies did not bind to the cells (Table 1).

**Table 1:** Antibody cell surface affinity.

	Anti-Her2 Cys-ADC	Anti-Her2 position 1-pAF	Anti-Her2 position 1-ADC	Anti-Her2 position 2-ADC	Anti-Her2 (wild type)	Anti-CD22 position 1-ADC	Anti-CD22 (wild type)
Bmax	1272	1353	1262	1668	1497	3.5	3.7
Kd (nM)	1.5	2.1	1.9	1.7	2.4	No binding	No binding

Antibody affinity was measured using HCC-1954 cells. The maximum binding and antibody affinity measurements were obtained for each antibody. HCC-1954 cells do not express CD22 therefore the anti-CD22 antibodies did not bind to the HCC-1954 cells.

The *in vitro* cytotoxicity of the anti-Her2 ADCs was evaluated using the Her2 expressing HCC-1954, NCI-N87 and MDA-MB-453 tumor cell lines and the MDA-MB-468 cell line, which does not express Her2. The anti-Her2 ADCs showed potent dose dependent *in vitro* cytotoxicity against the HCC-1954 and NCI-N87 cell lines and no *in vitro* cytotoxicity on the MDA-MB-468 cell line was observed. The anti-Her2 ADCs also showed potent dose dependent *in vitro* cytotoxicity on the Herceptin resistant MDA-MB-453 cell line while the unconjugated anti-Her2 antibodies were unable to inhibit MDA-MB-453 cell growth (Figure 4C). The anti-CD22 ADCs did not induce cytotoxicity on any of the cell lines except at the very highest concentration evaluated (10000 ng/ml).

### *In vivo* tumor efficacy studies

**Figure 5:**
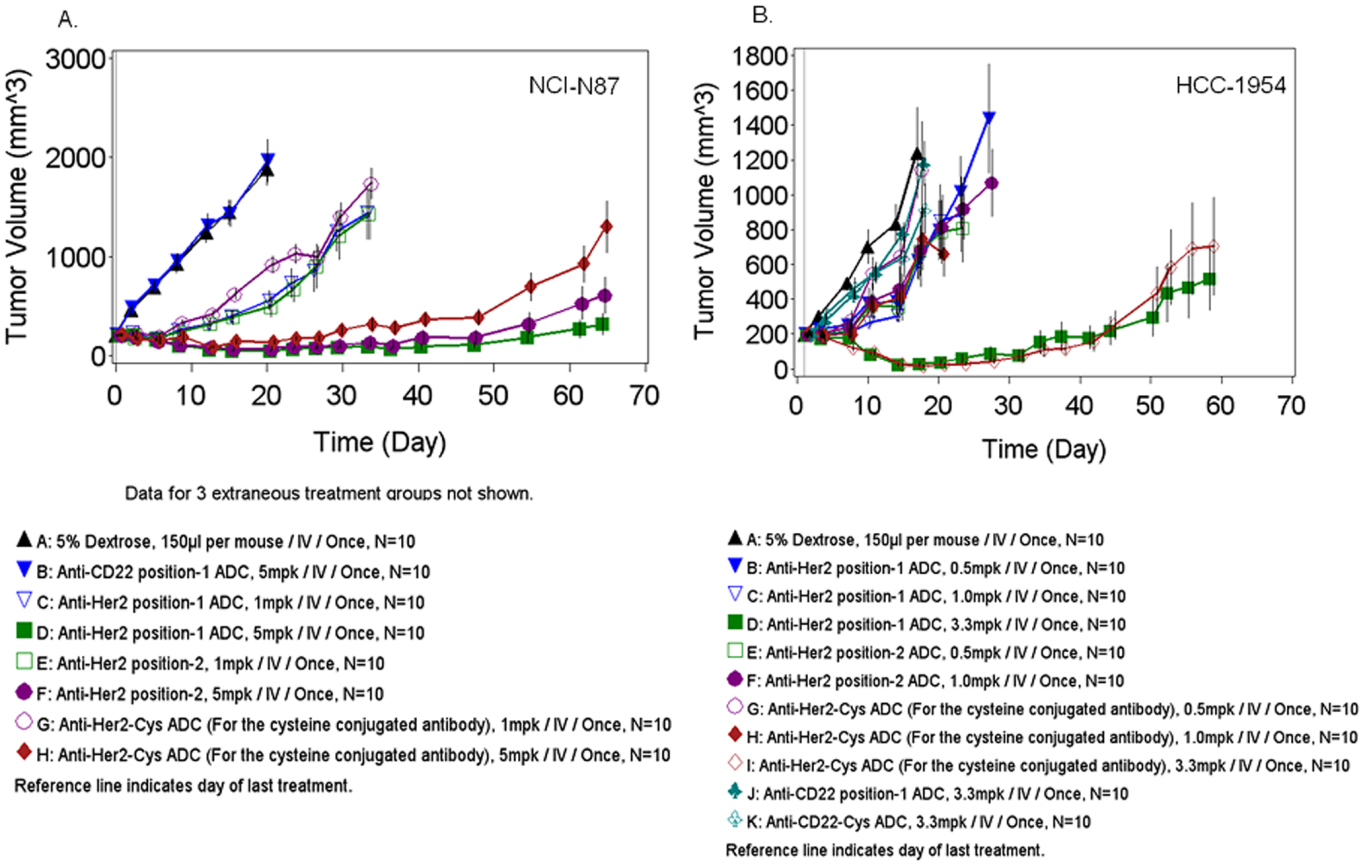
*In vivo* tumor efficacy studies. The *in vivo* efficacy of the anti-Her2 ADCs was evaluated in the NCI-N87 and HCC-1954 tumor xenografts. (A) SCID mice bearing NCI-N87 tumors were treated with a single, i.v. dose of anti-Her2 ADCs. (B) SCID mice bearing HCC-1954 tumors were treated with a single, i.v. dose of anti-Her2 ADCs.

The *in vivo* anti-tumor efficacy of the anti-Her2 position 1-ADC, anti-Her2 position 2-ADC against the anti-Her2 Cys-ADC in the NCI-N87 gastric cancer (Figure 5A) and HCC-1954 breast cancer (Figure 5B) xenografts grown in male and female SCID mice respectively.

NCI-N87 gastric tumor bearing mice were treated intravenously with a single injection of anti-Her2 position 1-ADC (1, 3 or 5 mg/kg) or anti-Her2 position 2-ADC (1, 3 or 5 mg/kg), anti-Her2 Cys-ADC (1, 3 or 5 mg/kg), the anti-CD22 position 1-ADC at 5 mg/kg or vehicle control (5% Dextrose), when the average tumor size reached approximately 200 mm^3^. All ADCs demonstrated statistically significant tumor efficacy compared to the anti-CD22 ADC (p<0.001) at all doses except for the anti-Her2 Cys-ADC at 1 mg/kg. Although the anti-Her2 position 1-ADC demonstrated efficacy at all doses, the efficacy of the 3 and 1 mg/kg doses were similar. In contrast, the anti-Her2 position-2-ADC showed no statistically significant difference in efficacy at the 3 and 5 mg/kg dose groups. Anti-Her2 Cys-ADC induced dose-dependent tumor reduction across the 3 doses evaluated (p<0.001). For purposes of clarity, the efficacy of the 3 mg/kg dose group is not shown in the figure.

HCC-1954 breast tumor bearing mice were treated intravenously with a single injection of anti-Her2 position 1-ADC(0.5, 1 or 3.3 mg/kg), anti-Her2 position 2-ADC (0.5 or 1 mg/kg), anti-Her2 Cys-ADC (0.5, 1 or 3.3 mg/kg) or the control ADCs (Anti-CD22 position 1-ADC or Anti-CD22-Cys-ADC at 3.3 mg/kg) or Vehicle (5% Dextrose) when the average tumor size reached approximately 200 mm^3^. As shown in Figure 5B, statistically significant tumor regression (P = 0.0012) was observed with anti-Her2 position 1-ADC and the anti-Her2 Cys-ADC at the highest dose tested (3.3 mg/kg) when compared to the control ADC. However, there was no statistically significant difference in efficacy between these two treatment groups. The specific anti-Her2 ADCs or the cysteine conjugated anti-Her2 ADC at the lower dose groups (0.5 and 1 mg/kg) did not show statistically significant efficacy compare to the control groups.

In these *in vivo* anti-tumor efficacy studies, the ADCs were well tolerated since no reduction in body weight or signs of distress were observed in any of the treatment groups (data not shown). In summary the anti-Her2 Cys-ADC, anti-Her2 position 1 and anti-Her2 position 2-ADCs showed dose-dependent anti-tumor efficacy in the HCC-1954 breast and NCI-N87 gastric xenograft models. The efficacy observed with anti-Her2 position 1 or anti-Her2 position 2-ADCs was comparable to the anti-Her2 Cys-ADC.

### Rat toxicokinetics

**Figure 6:**
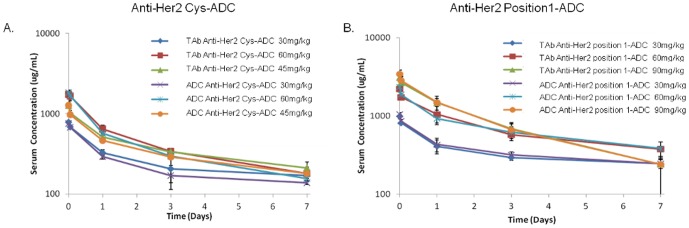
Rat toxicokinetics. Serum from rats dosed with 30, 45 or 60/kg of anti-Her2 Cys-ADC or 30, 60 or 90 mg/kg of anti-Her2 position 1-ADC were collected and the total amount of antibody (TAb) or ADC was measured via ELISA. (A) The pharmacokinetic profile of the anti-Her2 Cys-ADC was evaluated. (B) The pharmacokinetic profile of the anti-Her2 position 1-ADC was evaluated.

The serum concentrations of the anti-Her2 position 1-ADC and the anti-Her2 Cys-ADC were determined using the ADC and total antibody (TAb) ELISA assays. The serum concentration time profiles for anti-Her2 Cys-ADC and anti-Her2 position 1-ADC are shown in Figures 6A and 6B, respectively. The toxicokinetics of ADC and total antibody upon administration of anti-Her2 position 1-ADC and anti-Her2 Cys-ADC antibody followed a biphasic disposition with an initial rapid distribution phase followed a prolonged elimination phase. Maximal serum concentrations (C_max_) for ADC and TAb upon administration of anti-Her2 position 1-ADC and anti-Her2 Cys-ADC were at 5 minutes post i.v. injection. The TAb and ADC concentrations for both ADCs were similar and overlapping indicating minimal loss of the payload from the antibodies (Figures 6A and 6B).

ADC and TAb toxicokinetic parameters for animals were derived by non-compartmental analysis of the animal's concentration-time data. ADC and TAb TK parameters following i.v. administration of anti-Her2 position 1-ADC and anti-Her2 Cys-ADC are presented in (Table 2). The elimination half-life (T½ λz) for both antibodies, as measured by either ADC or TAb formats, decreased with increasing doses. However the area under the serum concentration curve [AUC_(last)_] and C_max_, for both ADC and TAb, showed dose-dependent increases. These early and limited data suggest almost dose linear toxicokinetics based on AUC and Cmax; however, definitive dose linearity could not be concluded due to the limited amount of data obtained in this exploratory study. The serum exposure for the anti-Her2 position 1-ADC was higher than the anti-Her2 Cys-ADC at all dose levels (Table 2).

**Table 2:** Rat toxicokinetics summary.

		Total Antibody			ADC		
Antibody	Dose (mg/kg)	T_1/2 (days)_	C_max (µg/ml)_	AUC _last(day*µg/ml)_	T_1/2 (days)_	C_max (µg/ml)_	AUC _last(day*µg/ml)_
Anti-Her2 position 1-ADC	30	9.12±2.19	1003±41.9	2418±168	7.89±1.32	1056±55.7	2554±178
Anti-Her2 position 1-ADC	60	4.36±0.495	2244±130	4978±865	4.94±0.812	2361±189	4959±604
Anti-Her2 position 1-ADC	90	2.40±0.871	3104±188	6153±838	2.31±0.734	3469±417	6214±974
Anti-Her2 Cys-ADC	30	7.31±2.03	781±19.1	1801±270	6.35±1.47	757±69.2	1581±228
Anti-Her2 Cys-ADC	45	4.91±0.871	1271±124	2726±182	4.60±0.926	1269±79.3	2429±167
Anti-Her2 Cys-ADC	90	3.13±0.852	1740±214	2938±314	3.04±0.885	1827±152	2696±300

Serum from rats dosed with 30, 45 or 60 mg/kg of anti-Her2 Cys-ADC or 30, 60 or 90 mg/kg of anti-Her2 position 1-ADC were collected and the total amount of antibody and ADC was measured via ELISA. The toxicokinetic parameters were calculated.

### Rat toxicology

A single injection of the anti-Her2 position 1-ADC was well tolerated up to 90 mg/kg. No early mortalities occurred. In comparison moribund sacrifices were required at doses of 45 and 60 mg/kg of anti-Her2 Cys-ADC. The cause of moribund condition was associated with moderate to marked body weight decrease (17–27%), general decline in appearance and treatment related findings in the liver (moderate hepatocyte necrosis and hypertrophy). There were no adverse clinical observations noted for the anti-Her2 position 1-ADC over the study period whereas rats treated with the anti-Her2 Cys-ADC were observed to have decreased skin turgor, decreased activity, urine staining, hunched appearance, pale ears, piloerection and/or rough haircoat.

**Figure 7:**
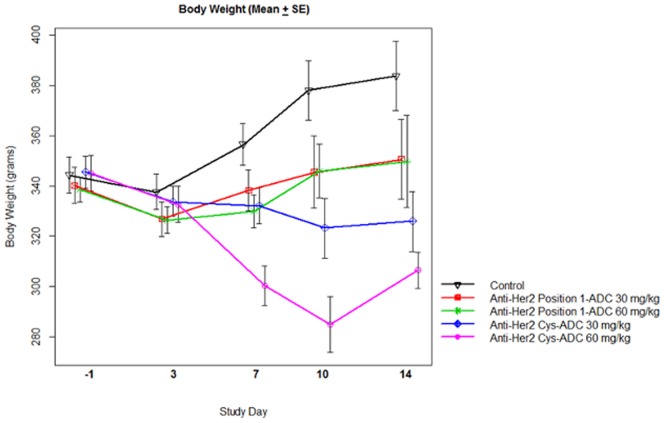
Rat body weights. Rat body weights were measured post single dose, i.v. administration of 30 or 60/kg of the anti-Her2 Cys or anti-Her2 position 1-ADC. Data represent Mean ± SE.

We evaluated the change in body weight between the treatment groups, as another measure of toxicity. ANOVA analysis was conducted on treatment groups with body weight measurements from day −1, the day before treatment, and day 10. The change in body weight on day 10 from day −1 was compared between the anti-Her2 position 1-ADC and anti-Her2 Cys-ADC treatment groups. The mean difference in the change of body weight for the 60 mg/kg group was 65.14 grams, p<0.0001 and the mean difference in the change of body weight for the 30 mg/kg group was 35.9 grams, p = 0.0006 (Figure 7). The decrease in body weight was generally dose-related for the anti-Her2 Cys-ADC groups and no significant body weight loss we observed for the anti-Her2 position 1-ADC treatment groups.

Changes in hematology and serum chemistry were compared between the treatment groups. On study day 4, anti-Her2 position 1-ADC and anti-Her2 Cys-ADC-related changes in hematology parameters included decreases in hemoglobin, and platelet counts; and increases in red cell distribution width (RDW), reticulocytes, white blood cell count (WBC), neutrophil counts, lymphocyte counts and monocyte counts compared to control (Table 3). These effects were more severe and present at lower dose levels in the anti-Her2 Cys-ADC groups compared to the anti-Her2 position 1-ADC groups. Anti-Her2 position 1-ADC and anti-Her2 Cys-ADC-related changes in serum chemistry parameters included increases in alanine aminotransferase (ALT), aspartate aminotransferase (AST), alkaline phosphatase, gamma glutamyl transferase (GGT), and triglycerides (Table 4). These changes in serum enzymes and lipid were attributed to the histopathologic findings in the liver. Albumin was decreased and globulin increased with a resultant decrease in albumin/globulin ratio (A/G). These findings combined with the increase in neutrophil counts indicated an acute phase response that was consistent with inflammation associated with the histologic findings in the liver and kidneys. BUN was increased, which was consistent with the histologic findings in the renal tubules. The effects were generally more severe and present at lower dose levels in the anti-Her2 Cys-ADC groups compared to anti-Her2 position 1-ADC.

**Table 3:** Hematology summary.

Parameter	Control	Anti-Her2 position 1-ADC	Anti-Her2 position 1-ADC	Anti-Her2 position 1-ADC	Anti-Her2 Cys-ADC	Anti-Her2 Cys-ADC	Anti-Her2 Cys-ADC
		30 mg/kg	60 mg/kg	90 mg/kg	30 mg/kg	45 mg/kg	60 mg/kg
Hemoglobin	14.7±0.2	13.4±0.5	11.9±1.0	11.9±1.0	12.4±0.7	12.6±0.5	10.8±0.9
RDW	11.9±0.3	14.4±0.5	15.6±1.2	17.5±0.5	15.0±0.6	18.6±2.9	21.4±3.3
Reticulocyte Count	2.23±0.24	4.14±1.02	4.18±1.16	6.57±1.21	4.30±0.86	7.42±2.39	7.63±1.22
Platelet Count	1158±190	1433±246	1239±514	879±532	1290±348	960±513	627±246
WBC	4.7±1.8	8.8±2.5	16.4±12.6	26.1±9.0	10.7±1.5	18.4±6.8	30.6±14.5
Neutrophil Count	688±304	2260±585	6674±4749	11799±4439	4122±817	8137±4616	13023±4330
Lymphocyte Count	3794±1237	5947±1951	8493±7481	12269±4713	5584±873	8264±2287	13719±8196
Monocyte Count	99±49	322±174	643±411	1356±797	425±155	1045±861	2991±19923

Comparison of selected hematology measurements between cysteine and site specifically conjugated Her2 ADCs. Hematology measurements were evaluated post single dose, i.v. administration of 30, 45 or 60 mg/kg of the anti-Her2 Cys or 30, 60 or 90 mg/kg of the anti-Her2 position 1-ADC. Data represent Mean ± SD.

**Table 4:** Rat serum chemistry summary.

Parameter	Control	Anti-Her2 position 1-ADC	Anti-Her2 position 1- ADC	Anti-Her2 position 1- ADC	Anti-Her2 Cys-ADC	Anti-Her2 Cys-ADC	Anti-Her2 Cys-ADC
		30 mg/kg	60 mg/kg	90 mg/kg	30 mg/kg	45 mg/kg	60 mg/kg
ALT	33±3	51±8	122±88	112±27	178±98	180±124	345±365
AST	108±28	223±38	697±770	619±294	848±351	732±381	2419±2853
Alkaline phosphatase	150±28	196±53	612±680	573±318	329±72	541±183	898±412
GGT	2±0	3±1	26±41	36±27	7±6	19±14	50±29
BUN	15±1	21±2	22±13	19±3	22±3	21±8	42±15
Globulin	2.3±0.1	3.0±0.2	3.6±0.5	3.9±0.4	3.8±0.4	3.6±0.2	3.2±0.4
Albumin	3.1±0.1	3.0±0.1	2.7±0.2	2.6±0.2	2.8±0.3	2.3±0.2	2.0±0.3
A/G	1.3±0.1	1.0±0.1	0.8±0.2	0.7±0.1	0.8±0.1	0.7±0.1	0.6±0.1
Triglycerides	53±14	52±14	92±41	216±69	61±16	190±186	195±57

Comparison of selected serum chemistry parameters (Day 15) between cysteine and site specifically conjugated Her2 ADCs. A panel of serum markers was evaluated post single dose, i.v. administration of 30 or 60 mg/kg or 90 mg/kg of the anti-Her2 Cys or 30, 45 or 60 mg/kg anti-Her2 position 1-ADC.

Gross and microscopic morphology differences were compared between the treatment groups. On day 15, anti-Her2 position 1-ADC and anti-Her2 Cys-ADC treatment related gross findings were seen in the reproductive tract and lymphoid tissue (Data not shown). Microscopic findings were observed in the kidney, liver, lung, spleen and testes. Soft and/or small testes were seen in both treatment groups at ≥30 mg/kg with secondary findings of small epididymis, prostate and seminal vesicles. Correlating degeneration of the seminiferous tubules was seen at ≥30 mg/kg for both test articles but the severity was least in the 30 mg/kg anti-Her2 position 1-ADC animals. Decreased absolute and relative testis, epididymis, and prostate weights were seen at ≥30 mg/kg with both test articles. Enlargement of the spleen, correlating to increased hemopoiesis, and lymph nodes were seen at ≥60 mg/kg anti-Her2 position 1-ADC and ≥30 mg/kg anti-Her2 Cys-ADC. Increased spleen weight was seen at ≥30 mg/kg of both test articles. Kidney tubule basophilia and protein casts were seen in a dose dependent pattern at ≥30 mg/kg for both test articles with increased weight at ≥60 mg/kg. Liver findings of necrosis were seen at ≥60 mg/kg for the anti-Her2 position 1-ADC and ≥30 mg/kg for the anti-Her2 Cys-ADC. Hypertrophy was seen at ≥30 mg/kg for both test articles, but there was an increased incidence and severity seen at 30 mg/kg dose of the anti-Her2 Cys-ADC. Increased liver weight was seen at ≥30 mg/kg of both test articles. Deposition of amphophilic flocculent material in the lungs and increased organ weight was seen at ≥30 mg/kg for both test articles. Increased absolute and relative adrenal gland weights were seen at ≥60 mg/kg of the anti-Her2 position 1-ADC and ≥30 mg/kg of the anti-Her2 Cys-ADC.

The results of this toxicology study demonstrate that the anti-Her2 position 1-ADC is better tolerated than the anti-Her2 Cys-ADC at comparable doses. A single dose up to 90 mg/kg of anti-Her2 position 1-ADC was not associated with early mortality, but there were 4 test article related early mortalities in animals dosed with the anti-Her2 Cys-ADC at lower doses (3 at 60 mg/kg and 1 at 45 mg/kg). All dose levels of the anti-Her2 Cys-ADC were associated with body weight loss over the 15 day study period, whereas only the 90 mg/kg treated group of the anti-Her2 Cys-ADC lost weight (data not shown). Qualitatively, similar changes were noted for clinical and anatomic pathology after treatment with either the anti-Her2 position 1-ADC or the anti-Her2 Cys-ADC, but were generally seen at lower doses and were more severe for the anti-Her2 Cys-ADC test article, even with the reduced systemic exposure (C_max_ and AUC) as compared to the anti-Her2 position 1-ADC.

## Discussion

ADCs have undergone a series of advancements starting with improvements in the potency of the payload, drug linker technology and antibody engineering. These improvements have resulted in potentially safer and more effective ADCs.

One of the early ADCs to enter clinical development was cBR96-Dox (SGN-15). cBR96-Dox was a chimeric anti-Lewis Y antibody conjugated via a hydrazone linker to Doxorubicin [13]. The results from the clinical trial suggested that improvements in the potency of the payload, linker design and antibody engineering would result in improved clinical activity [14]. In 2000, Mylotarg, a humanized IgG4 anti-CD33 antibody (hP67.6) conjugated to Calicheamicin via a hydrazone linker on lysine residues, was the first ADC to be approved by the FDA for the treatment of acute myeloid leukemia (AML) patients. Calicheamicin has potent *in vitro* cytotoxicity against proliferating cells, but its physical properties produced significant CMC challenges resulting in approximately 50% of the ADC preparation containing unconjugated antibody [15]. Although several possibilities exist to explain Mylotarg's lack of clinical benefit, one distinct possibility is the high percentage of unconjugated anti-CD33 antibody may have reduced Mylotarg's potency resulting in a lack of clinical benefit. The recent approval of SGN-35 (Adcetris), an anti-CD30 Auristatin ADC for the treatment of Hodgkin's lymphoma and Trastuzumab Emtansine (T-DM1), an anti-Her2/ErbB2 ADC for the treatment of malignant breast cancer, provides evidence of these improvements.

The current set of ADCs in clinical development use ADC payloads that are attached to protease cleavable, acid labile or non cleavable linkers. These ADC payloads and linkers are covalently attached to the amines on lysines or the thiols on cysteines which results in a heterogeneous mixture of antibodies containing 0 to 8 drugs per antibody [14]. ADCs which contain 8 drugs per antibody are less stable in circulation as compared to ADCs which have a lower number of drugs per antibody. As the number of drugs per antibody decreases, the pharmacokinetic properties of the ADC improve [16]. The instability of the ADCs with a higher DAR may result in the systemic release of drug payload, which may be responsible for the non target-related toxicities reported for various ADCs. Therefore the goals for the next generation of ADCs are to reduce the number of drugs per antibody, which will improve the pharmacokinetic properties of the ADC; to improve the stability of the ADC payload, which will reduce the non target related toxicities; and improve the CMC properties of the ADC while maintaining the *in vivo* anti tumor efficacy and the *in vitro* cytotoxicity of the ADC.

The direct incorporation of the non-natural amino acid, Para-acetyl phenylalanine (pAF) at specific sites on an anti-Her2 antibody, allows for the site specific incorporation of two drugs per antibody [11]. This is similar to what was reported for modified version of T-DM1 (ThioTMab) where the specific incorporation of cysteines into Herceptin resulted in a homogeneous preparation containing 1.8 drugs per antibody [10]. The lack of unconjugated antibody in the preparations offers a distinct advantage over the ADCs where the ADC payload is conjugated to antibody's free cysteine or lysine residues.

The conjugation chemistry used to attach the drug payload A to pAF generates an oxime bond, which has been reported to be very stable [17]. The oxime bond used to conjugate payload A to pAF may be more stable than the maleimide used to conjugate the Auristatin drug payloads to the thiols on cysteines. The use of maleimide for the conjugation of ADC payloads to thiols has been reported to result in the transfer of the drug payload from the antibody to the free thiol found on HSA through a retro-Michael reaction [18]. We show that the anti-Her2 Cys-ADC transferred substantially more ADC payload to HSA than the anti-Her2 position 1-ADC or the anti-Her2 position 2-ADC. These data provide further evidence that ADC payloads, conjugated to antibodies via thiols results in the loss of the ADC payload to HSA.

The *in vitro* cytotoxicity data show the anti-Her2 Cys-ADC, the anti-Her2 position 1 and the anti-Her2 position 2-ADCs had similar cytotoxicity against Her2 expressing HCC-1954 and NCI-N87 cells and were inactive against the MDA-MB-468 cells, which do not express Her2. These data show that cell surface Her2 expression is required for the *in vitro* cytotoxicity of the anti-Her2 ADCs. We further showed that the anti-Her2 ADCs have similar cytotoxicity against Herceptin resistant MDA-MB-453 cells, while the unconjugated anti-Her2 antibodies were not cytotoxicity on MDA-MB-453 cells. These data are consistent with data showing that T-DM1 can inhibit the growth of Herceptin resistant tumors, JIMT-1, MDA-MB-453 and UACC-893 [7].

The anti-Her2 Cys-ADC, the anti-Her2 position 1-ADC and the anti-Her2 position 2-ADC showed dose dependent inhibition of the HCC-1954 and NCI-N87 tumors *in vivo*. Although the anti-Her2 position 1-ADC and the anti-Her2 position 2-ADC did not provide superior *in vivo* tumor efficacy as compared to the anti-Her2 Cys-ADC in HCC-1954 tumors, the anti-Her2 position 1 and anti-Her2 position 2-ADCs contained only half of the amount of drug as the anti-Her2 Cys-ADC. A similar observation was reported showing ThioTMab had comparable *in vivo* tumor efficacy as compared to T-DM1 yet the ThioTMab contained half the amount of drug payload as the lysine conjugated T-DM1 [10]. The potent *in vivo* anti-tumor efficacy of the site specifically conjugated ADCs could be the result of having a higher percentage of drug conjugated antibody, no unconjugated antibody and improved pharmacokinetic properties.

Although the site specific anti-Her2 ADCs showed comparable *in vitro* cytotoxicity and *in vivo* tumor efficacy as the cysteine conjugated anti-Her2 ADC, the rat toxicology study revealed significant differences in the toxicology profiles of the anti-Her2 position 1-ADC and the anti-Her2 Cys-ADC.

The rats treated with a single, i.v. injection of 30 or 60 mg/kg anti-Her2 Cys-ADC experienced substantial weight loss while the rats treated with 30 or 60 mg/kg of the anti-Her2 position 1-ADC did not lose weight. Furthermore, rats treated with 60 mg/kg of the anti-Her2 Cys-ADC experienced a decrease in platelets while a decrease in the number of platelets was only observed when the rats were treated with 90 mg/kg of the anti-Her2 position 1-ADC. These data are similar to those reported for T-DM1 where decreases in body weight and platelets were observed at 47 mg/kg [10].

The rat serum chemistry panel shows a substantial dose dependent increase in the serum levels of the AST and ALT which are associated with liver damage, in the rats treated with anti-Her2 Cys-ADC. In contrast, rats treated with the anti-Her2 position 1-ADC, had substantially lower increase of these proteins. These data show the anti-Her2 position 1-ADC exhibited an improved safety profile as compared to the anti-Her2 Cys-ADC. This may be due, in part, to a higher amount of ADC payload being transferred from the anti-Her2 Cys-ADC to HSA. The transfer of the ADC payload from the ADC to HSA could result in increased drug half life and non-target related toxicities. The improved safety profile of the anti-Her2 position 1-ADC, along with the higher systemic exposures of the anti-Her2 position 1-ADC and potent *in vivo* tumor efficacy may result in an improved therapeutic index in the clinical trials.

The rat toxicokinetic data show higher AUCs and Cmax values for the anti-Her2 position 1-ADC as compared to the anti-Her2 Cys-ADC. These data, combined with data showing undetectable transfer of the ADC payload to HSA, suggest that conjugation via an oxime bond is more stable than conjugating via thiols.

Although site specifically conjugated ADCs, such as anti-MUC16 Thiomab, have been reported to be more stable than the traditional cysteine conjugated ADCs, the site specific conjugation of a drug payload using maleimide conjugation can result in the *in vitro* and *in vivo* loss of drug payload [15]. We did not observe significant loss of payload A from the anti-Her2 payload 1-ADC or the anti-Her2 payload 2-ADCs in the 28 day *in vitro* serum stability studies yet loss of the anti-MUC16 Thiomab payload, monomethyl auristatin E (MMAE) was observed after 4 days in serum [15]. The selection of the appropriate site for conjugation of the drug payload to the antibody is critical to the stability of the ADC [18]. The careful selection of the ADC drug payload along with the proper selection of the site used to incorporate the ADC payload appear to be vital for the efficacy and safety profile for an engineered ADC.

The clinical experience gained from ADCs that have been discontinued, may result in substantial improvements in the next generation of ADCs. The clinical development of two ADCs, Gemtuzumab Ozogamicin (Mylotarg) and AVE9633, which were directed against CD33 have been discontinued, yet a new ADC targeting CD33 (SGN-CD33A) is in preclinical development. SGN-CD33A utilizes the site specific incorporation of a pyrrolobenzodiazepine (PBD) dimer as the drug payload on engineered cysteine residues via a protease cleavable linker [19]. The combination of the potent PBD payload conjugated to two engineered cysteines on the CD33 antibody may provide an advantage over the previous anti-CD33 ADCs. A possible disadvantage of SGN-CD33A is that the PBD payload may be liberated from the antibody via a retro-Michael reaction, which could result in non target related toxicities. The use of the more stable oxime bond to site specifically conjugate ADC payloads to antibodies should result in improved pharmacokinetics and diminished non target related toxicology risk.

We report the results from the first direct preclinical comparison of a cysteine conjugated ADC with non-natural amino acid site specific ADCs. Our data show that the site specific incorporation of a non-natural amino acid into the heavy chain of an anti-Her2 antibody combined with the stability of the oxime bond results in a potent and stable ADC. We further show that the site specific non-natural amino acid anti-Her2 ADC has superior CMC and toxicology profiles than a cysteine conjugated anti-Her2 ADC. We believe that the site specific incorporation of an ADC payload, using oxime conjugation, may provide an improved therapeutic window which should result in clinical benefit to cancer patients.

## References

[1] Goldmacher VS, Kovtun YV (2011) Antibody-drug conjugates: using monoclonal antibodies for delivery of cytotoxic payloads to cancer cells. Ther Deliv 2: 397–416. 22834009 10.4155/tde.10.98

[2] Meden H, Kuhn W (1997) Overexpression of the oncogene c-erbB-2 (HER2/neu) in ovarian cancer: a new prognostic factor. Eur J Obstet Gynecol Reprod Biol 71: 173–179. 9138962 10.1016/s0301-2115(96)02630-9

[3] Slamon DJ, Clark GM, Wong SG, Levin WJ, Ullrich A, et al (1987) Human breast cancer: correlation of relapse and survival with amplification of the HER-2/neu oncogene. Science 235: 177–182. 3798106 10.1126/science.3798106

[4] Papazisis KT, Habeshaw T, Miles DW (2004) Safety and efficacy of the combination of trastuzumab with docetaxel for HER2-positive women with advanced breast cancer. A review of the existing clinical trials and results of the expanded access programme in the UK. Int J Clin Pract 58: 581–586. 15311558 10.1111/j.1368-5031.2004.00203.x

[5] Hurvitz SA, Kakkar R (2012) The potential for trastuzumab emtansine in human epidermal growth factor receptor 2 positive metastatic breast cancer: latest evidence and ongoing studies. Ther Adv Med Oncol 4: 235–245. 22942906 10.1177/1758834012451205 PMC3424498

[6] Hubalek M, Brunner C, Mattha K, Marth C (2010) Resistance to HER2-targeted therapy: mechanisms of trastuzumab resistance and possible strategies to overcome unresponsiveness to treatment. Wien Med Wochenschr 160: 506–512. 20972709 10.1007/s10354-010-0838-6

[7] Barok M, Tanner M, Koninki K, Isola J (2011) Trastuzumab-DM1 causes tumour growth inhibition by mitotic catastrophe in trastuzumab-resistant breast cancer cells in vivo. Breast Cancer Res 13: R46. 21510863 10.1186/bcr2868 PMC3219209

[8] Lewis Phillips GD, Li G, Dugger DL, Crocker LM, Parsons KL, et al (2008) Targeting HER2-positive breast cancer with trastuzumab-DM1, an antibody-cytotoxic drug conjugate. Cancer Res 68: 9280–9290. 19010901 10.1158/0008-5472.CAN-08-1776

[9] Guha M (2012) T-DM1 impresses at ASCO. Nat Biotech 30: 728–728.

[10] Junutula JR, Flagella KM, Graham RA, Parsons KL, Ha E, et al (2010) Engineered thio-trastuzumab-DM1 conjugate with an improved therapeutic index to target human epidermal growth factor receptor 2-positive breast cancer. Clin Cancer Res 16: 4769–4778. 20805300 10.1158/1078-0432.CCR-10-0987

[11] Axup JY, Bajjuri KM, Ritland M, Hutchins BM, Kim CH, et al (2012) Synthesis of site-specific antibody-drug conjugates using unnatural amino acids. Proc Natl Acad Sci U S A 10.1073/pnas.1211023109 PMC3479532 22988081

[12] Iman WJ, Ca RL (1981) Rank transformations as a bridge between parametric and nonparametric statistics. The American Statistician 35: 124–129.

[13] Trail PA, Willner D, Lasch SJ, Henderson AJ, Hofstead S, et al (1993) Cure of xenografted human carcinomas by BR96-doxorubicin immunoconjugates. Science 261: 212–215. 8327892 10.1126/science.8327892

[14] Valliere-Douglass JF, McFee WA, Salas-Solano O (2012) Native intact mass determination of antibodies conjugated with monomethyl Auristatin E and F at interchain cysteine residues. Anal Chem 84: 2843–2849. 22384990 10.1021/ac203346c

[15] Xu K, Liu L, Saad OM, Baudys J, Williams L, et al (2011) Characterization of intact antibody-drug conjugates from plasma/serum in vivo by affinity capture capillary liquid chromatography-mass spectrometry. Anal Biochem 412: 56–66. 21216214 10.1016/j.ab.2011.01.004

[16] Hamblett KJ, Senter PD, Chace DF, Sun MM, Lenox J, et al (2004) Effects of drug loading on the antitumor activity of a monoclonal antibody drug conjugate. Clin Cancer Res 10: 7063–7070. 15501986 10.1158/1078-0432.CCR-04-0789

[17] Crisalli P, Kool ET (2013) Water-soluble organocatalysts for hydrazone and oxime formation. J Org Chem 78: 1184–1189. 23289546 10.1021/jo302746p PMC3562402

[18] Shen BQ, Xu K, Liu L, Raab H, Bhakta S, et al (2012) Conjugation site modulates the in vivo stability and therapeutic activity of antibody-drug conjugates. Nat Biotechnol 30: 184–189. 22267010 10.1038/nbt.2108

[19] Kung Sutherland MS, Walter RB, Jeffrey SC, Burke PJ, Yu C, et al (2013) SGN-CD33A: a novel CD33-targeting antibody-drug conjugate using a pyrrolobenzodiazepine dimer is active in models of drug-resistant AML. Blood 122: 1455–1463. 23770776 10.1182/blood-2013-03-491506

